# Pentagalloyl Glucose, a Major Compound in Mango Seed Kernel, Exhibits Distinct Gastroprotective Effects in Indomethacin-Induced Gastropathy in Rats *via* Modulating the NO/eNOS/iNOS Signaling Pathway

**DOI:** 10.3389/fphar.2022.800986

**Published:** 2022-02-04

**Authors:** Mona F. Mahmoud, Mohamed Nabil, Rehab A. Hasan, Assem M. El-Shazly, Mohamed A. El-Ansari, Mansour Sobeh

**Affiliations:** ^1^ Department of Pharmacology and Toxicology, Faculty of Pharmacy, Zagazig University, Zagazig, Egypt; ^2^ Pharmaceutical and Fermentation Industries Development Center (PFIDC), City of Scientific Research and Technological Applications (SRTA-City), New Borg El-Arab, Alexandria, Egypt; ^3^ Pharmacology Department, Faculty of Pharmacy, Deraya University, New Mina, Egypt; ^4^ Department of Histology, Faculty of Medicine for Girls, Al Azhar University, Cairo, Egypt; ^5^ Department of Pharmacognosy, Faculty of Pharmacy, Zagazig University, Zagazig, Egypt; ^6^ Phytochemistry and Plant Systematics Department, National Research Centre, Cairo, Egypt; ^7^ AgroBioSciences, Mohammed VI Polytechnic University, Ben-Guerir, Morocco

**Keywords:** indomethacin, pentagalloyl glucose, gastric ulcer, iNOS, eNOS, PECAM-1, VEGF

## Abstract

Gastric ulcers are a common health disorder that affect up to 10% of the world’s population. The gastroprotective potential of pentagalloyl glucose (PGG) against indomethacin-induced ulcer in rats and the possible underlying mechanisms were investigated. Gastric ulceration was induced by indomethacin (single dose, 60 mg/kg). Pretreatment with PGG (100 or 200 mg/kg, orally) for 8 days prior to the administration of indomethacin furnished significant reductions in gastric mucosal lesions as well as a significant increase in mucus concentration. Also, PGG significantly declined the elevations in gastric mucosal MDA, TNF-α, IL-6, PECAM-1, VEGF, and iNOS expression. It also mitigated the decrease in GSH and GPx and eNOS expression observed with indomethacin. The protective effects furnished by PGG were comparable to that of famotidine. The obtained results suggested that the anti-ulcer effects of PGG are mediated by increasing mucus production, scavenging free radicals, decreasing inflammation, and attenuating the NO/NOS signaling in favor of eNOS. To sum up, PGG could provide a potential therapy for gastric ulcer after evaluating its efficacy and effectiveness.

## 1 Introduction

Gastric ulcers, also known as peptic ulcers, are painful sores that develop in the stomach lining when the mucus layer is reduced or damaged. Gastric ulcer etiology includes several factors, such as excessive reactive oxygen/nitrogen species production, alcohol and spicy food intake, tobacco, radiation therapy, excessive use of NSAIDs, bile reflux, and *Helicobacter pylori* infection. The treatment of ulcer could be complicated because the pathology is multifactorial and includes antioxidant activity, inflammation, and angiogenesis, among others. Medications such as histamine H2-receptor antagonists and proton pump inhibitors are effectively used to treat gastric ulcers; however, they are associated with severe side effects. Therefore, there is a need to develop safe, natural, and multitarget agents (Ramakrishnan and Salinas, 2007; Lau et al., 2008; Mahmoud et al., 2021).

Medicinal plants that shape a major segment of flora have been used over centuries by mankind owing to their beneficial curing properties against diverse diseases and pathological conditions. These plants have been providing indigenous precursors and raw materials for medicine and drug industry all over the world. Natural polyphenols are the most abundant antioxidants present in a large number of foods of plant origin, such as fruits, vegetables, cereals, dry legumes, and beverages. Polyphenols exhibit a wide spectrum of biological activities and diverse chemical structures starting from simple phenolic compounds, such as phenolic acids and flavonoids, to oligomer tannins (Van Wyk and Wink, 2015; Ardalani et al., 2020). Mangiferin, a xanthonoid from *Mangifera indica* as well as from polyphenol-rich extract from leaf extracts of mango, exhibited gastroprotective activities highlighting the therapeutic activities of the plant (Carvalho et al., 2007; Severi et al., 2009)*.*

The polyphenolic compound pentagalloyl glucose (PGG), found in numerous herbs, fruits, and by-products, among them mango seed kernel, is a potent antioxidant belongs to tannins. PGG demonstrated robust anti-inflammatory, antiviral, antimicrobial, antidiabetic, and antitumor activities (Patnaik et al., 2019). A recent study highlighted the ability of PGG to inhibit cell viability and cell growth in triple-negative breast cancer through the inhibition of MAPK1 and ƙBKE genes and the protein expression of MAPK and IƙBKE. PGG also reduced the expression of human proinflammatory cytokines GRO and GRO-α/CXCL1 (Mendonca et al., 2021). We previously isolated PGG (1,2,3,4,6-penta-*O*-*β*-D-galloyl glucose) from *Mangifera indica* L. seed kernels and comprehensively characterized its structure using mass spectrometry and NMR techniques and highlighted its promising larvicidal activities against the deadliest insect *Culex pipiens* (Emam et al., 2022). Furthermore, a recent study highlighted the potential antiviral activities of PGG against COVID-19 *via* blocking the fusion of SARS-CoV-2 spike-RBD to hACE2 receptors (Chen et al., 2021).

Herein, we investigated the protective effects of PGG on indomethacin-induced gastric ulcer in rats. We also explored a wide array of biochemical markers, including inflammatory cytokines, nitrosative stress, and oxidative stress. Additionally, we researched the interactions of PGG, through molecular docking, with two prostaglandin receptors, namely, EP3 and EP4 that are crucially involved in PGE2 gastroprotective action.

## 2 Material and Methods

### 2.1 Compound Isolation and Characterization

*Mangifera indica* L. seed kernels were crushed into small pieces and macerated with distilled water for 24 h and filtered, and the combined extract was reduced under pressure till dryness using Rotavapor^®^ (Heizbad Hei-VAP, Heidolph, Germany). The combined extract was fractionated using ethyl acetate, and the obtained fraction was thoroughly cleaned using petroleum ether yielding gallotannin fraction. The latter was chromatographed over Sephadex LH-20 column (Pharmacia Co., Uppsala, Sweden) using methanol and prep-PC (Whatman 3 MM 46 × 57 cm) yielding PGG as a major compound. PGG identification was performed as previously described (Emam et al., 2022).

### 2.2 Animals

Male Wistar rats (200–250 g) had been purchased from the Faculty of Veterinary Medicine, Zagazig, Egypt. These rats were acclimatized to the animal house conditions (temperature of 25°C ± 2°C, with a light/dark cycle of 12 h/12 h) for 1 week before the study. Prior to administration of indomethacin, animals had free access to chow and tap water.

### 2.3 Experimental Design

The rats were randomly assigned into five groups (*n* = 6) as follows. Group 1 (control group) and group 2 received 2 ml/kg/day vehicle (5% Tween 80) for 8 days. Group 2 (ulcer group) was orally gavaged with a single dose of indomethacin (60 mg/kg, b.w.) (Nile Company for Pharmaceutical and Chemical Industries, Cairo, Egypt) 60 mg/kg after 24 h fasting (Slomiany and Slomiany, 2005). Groups 3–5 received 100 and 200 mg/kg of PGG (PGG 100, PGG 200)*,* and 10 mg/kg famotidine (Amoun pharmaceutical company, Cairo, Egypt) suspended in 2 ml of 5% Tween 80, respectively, for 8 consecutive days followed by single oral dose of indomethacin (60 mg/kg, b.w.) at the eighth day. Six h after the last treatment, the rats were killed by decapitation. The stomachs were excised out of each rat, opened, and washed with normal saline for estimating ulcer indices. The stomachs were divided into two parts: one part was fixed in 10% neutral buffered formalin and coated in paraffin for the histological examinations and the other part was frozen in liquid nitrogen and kept at −80°C for biochemical measurements. The doses of PGG were chosen based on a previous study of Moharram et al. (2017). Famotidine dose was selected based on a previous study of Campos-Vidal et al. (2021).

### 2.4 Determination of Gastric Ulcer Index

The stomachs from different groups were opened along their greater curvature, cleaned with cold normal saline, blotted between two filter papers to dry them, and placed on a cardboard for gross examination of ulcers. Digital images of the stomachs were taken. The photomacrographs were analyzed using ImageJ software (Wayne Rasband, MA, United States) to assess ulceration area according to Szabo and Hollander (Szabo and Hollander, 1989). The ulcer index was estimated using the following formula: ulcer index = [ulcerated area/total stomach area] × 100.

### 2.5 Determination of Gastric Mucosal Glycoproteins

For histochemical studies of gastric mucosal glycoprotein content, the tissues were stained with the Periodic acid–Schiff (PAS) technique with hematoxylin counterstaining (McManus, 1948; Al Batran et al., 2013). The slides were examined under a light microscope (Primo star, ZEISS, China). The photomicrographs were captured using a camera (Axiocam ERc 5s, ZEISS, China) at the Histology Department, Faculty of Medicine for Girls, Al Azhar University, Egypt.

### 2.6 Histological Examination of Gastric Lesions

For routine histological study, after proper fixation of the fundus of the stomach in 10% formaldehyde, it was dehydrated in an alcohol series of 100, 90, 70, and 50%, cleared in xylene, infiltrated, and embedded in paraffin and then sectioned (5 µm thick) using a rotary microtome (LEICA RM 2125, United Kingdom). They were further deparaffinized, stained with hematoxylin and eosin (H&E), and examined under a light microscope (Primo star, ZEISS, China) and photographed using a camera (Axiocam ERc 5s, ZEISS, China) (Bancroft and Gamble, 2008). The results were graded based on a scoring system (Sidahmed et al., 2019) as follows: epithelial cell loss (score 0–3), edema in submucosa (score: 0–4), hemorrhagic damage (score: 0–4), and the presence of inflammatory cells (score: 0–3). This scoring was performed using a light microscope (Primo star, ZEISS, China).

### 2.7 Immunohistochemical Studies

The stomach sections were processed and immune-stained using the peroxidase-labeled streptavidin–biotin method for iNOS and eNOS. The primary antibodies used in the present study were as follows: iNOS polyclonal antibodies (diluted 1:100; Abcam, Cat: ab15323, Cambridge, United Kingdom) and eNOS polyclonal antibodies (diluted 1:100; Abcam, Cat: ab5589, Cambridge, United Kingdom) (Wang et al., 2005). The secondary antibody used was anti-rabbit immunoglobulin (Ig, Santa Cruz Inc., Santa Cruz, CA, United States) conjugated with biotin. The positive slide was provided by the manufacturer. Negative control sections were prepared with omission of the primary antibody.

### 2.8 Morphometric Study

Morphometric study was performed using a Leica light microscope MDLSD coupled to a Leica digital camera transferred to the screen using a computerized image analyzer Leica Q500 MC program (Leica Microsystems Ltd., Cambridge, United Kingdom). The data were calibrated automatically to convert the measurement units (pixels) produced by the image analyzer program into actual micrometer units. Ten different non-overlapping randomly selected fields from a slide of each rat in all different experimental groups were examined to evaluate the following mean area percentage of PAS-positive histochemical reaction in PAS-stained sections at a magnification of 200; optical density of positive immunostaining for iNOS and eNOS was measured within and around the ulcer area at a magnification of 200 (Mahmoud et al., 2021).

### 2.9 Determination of Gastric Oxidative Stress

Gastric lipid peroxidation product, MDA, Gpx activity, and GSH were determined as reported previously (Mahmoud et al., 2021).

### 2.10 Determination of Gastric Inflammation

The levels of IL-6 and TNF-α in the gastric tissue lysate were determined using Sigma-Aldrich ELISA kits (Catalog numbers RAB0480 and RAB0311, respectively) according to the manufacturer’s instructions.

### 2.11 Western Blot Analysis

The stomach tissues were dissected and crushed in ice-cold Nonidet-P40 (NP40) buffer with the addition of 1% protease inhibitor cocktail (Boster Biological Technology, Pleasanton, CA, United States, Catalog number: AR1182). The supernatants were recovered after centrifuging the samples. The Bradford technique was used to determine protein content. Ten percent SDS-PAGE was used to separate tissue proteins, which were then transferred to nitrocellulose membranes using the Trans-Blot^®^ semi-dry transfer cell (Bio- Rad, Hercules, CA, United States) at 20 v for 15 min. After blocking with 1 × Tris-buffered saline/0.1% Tween 20 (TBST) with 5% non-fat dry milk for 1 h, rabbit polyclonal antibodies against VEGF (Catalog no: CAB12303; Assay genie, Dublin, Ireland; dilution, 1:500); primary rabbit polyclonal antibodies against PECAM-1 (Catalog no: PA5-96055; Invitrogen, Thermo Fisher Scientific corporation, Massachusetts, United States; dilution, 1:1,000); and primary rabbit monoclonal antibodies against β-actin (Catalog no: M01263; Boster Biological Technology, Pleasanton, CA, United States; diluted at 1:1,000) were all identified by the antibodies used (Sigma-Aldrich, United States). The membranes were subjected to goat anti-rabbit secondary antibodies (Catalog no: 5,220–0,308; SeraCare Life Sciences Inc., Massachusetts, United States; dilution, 1:3,000) for 2 h after being washed with 1 × TBST. Densitometrical analysis of protein bands was operated using Image Studio Lite software (LI-COR Biosciences, Nebraska, United States), and the protein expression was detected in consideration to its β-actin normalization.

### 2.12 Statistical Analysis

The data are presented as a mean standard error of the mean (S.E.M.). Student’s t-test and one-way ANOVA were used to perform statistical comparisons, which were then followed by Tukey’s multiple comparisons test. The severity scores of gastric injuries among different groups were performed by one-way analysis of variance (ANOVA), followed by Dunnett’s test. To illustrate statistical significance, a *p* value less than 0.05 was used. GraphPad Prism, version 8, was used to perform statistical calculations (GraphPad software Inc., La Jolla, CA, United States).

## 3 Results and Discussion

### 3.1 Effect of PGG on Indomethacin-Induced Ulcer Index

**FIGURE 1:**
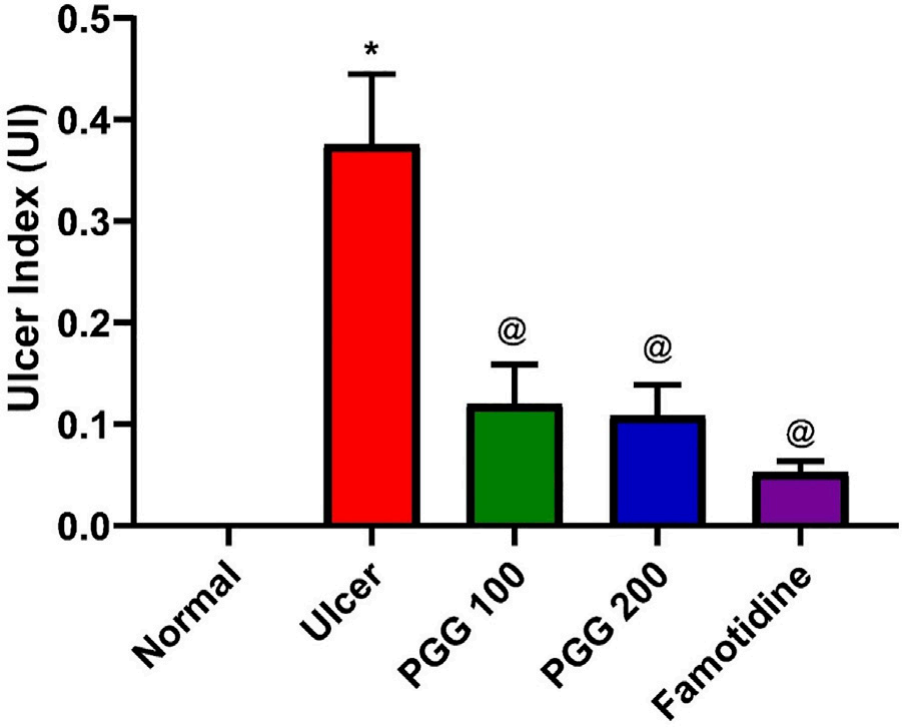
Effect of pentagalloyl glucose (PGG) and famotidine on the ulcer index in indomethacin-induced ulcer in rats. Values are means of six rats ± S.E.M; as compared with normal (*) and indomethacin-treated (^@^) groups (one-way ANOVA followed by Tukey’s multiple comparison tests), *p* < 0.05.

Peptic ulcer is a debilitating global health problem. It develops when the usual balance between the aggressive factors (acid and pepsin) and the defensive processes, such as mucus production, bicarbonate, mucosal turnover, and blood flow (mucosal barrier), is disrupted (Piper and Stiel, 1986). When compared to other NSAIDs, indomethacin is a potent NSAID widely used in clinical practice. Because it has a higher ulcerogenic potency than other NSAIDs, it is commonly utilized for the induction of gastric ulcer in experimental animals (Suleyman et al., 2010). Similar to previous studies, the current study also found that indomethacin induced gastric ulceration and mucosal damage as it increased the ulcer index (Mahmoud et al., 2021; Ahmed et al., 2021; Nabil et al., 2021). PGG inhibited the indomethacin-induced gastric injury as presented by low values of ulcer index, and the effect of PGG was comparable to that shown by famotidine (Figure 1). This study confirmed the previous study findings that PGG and famotidine suppressed gastric ulceration induced by aspirin in rats by increasing PGE2 expression, antioxidant, anti-inflammatory, and antisecretory effects (Moharram et al., 2017).

### 3.2 Effect of PGG on Gastric Mucosal Glycoprotein Content

**FIGURE 2:**
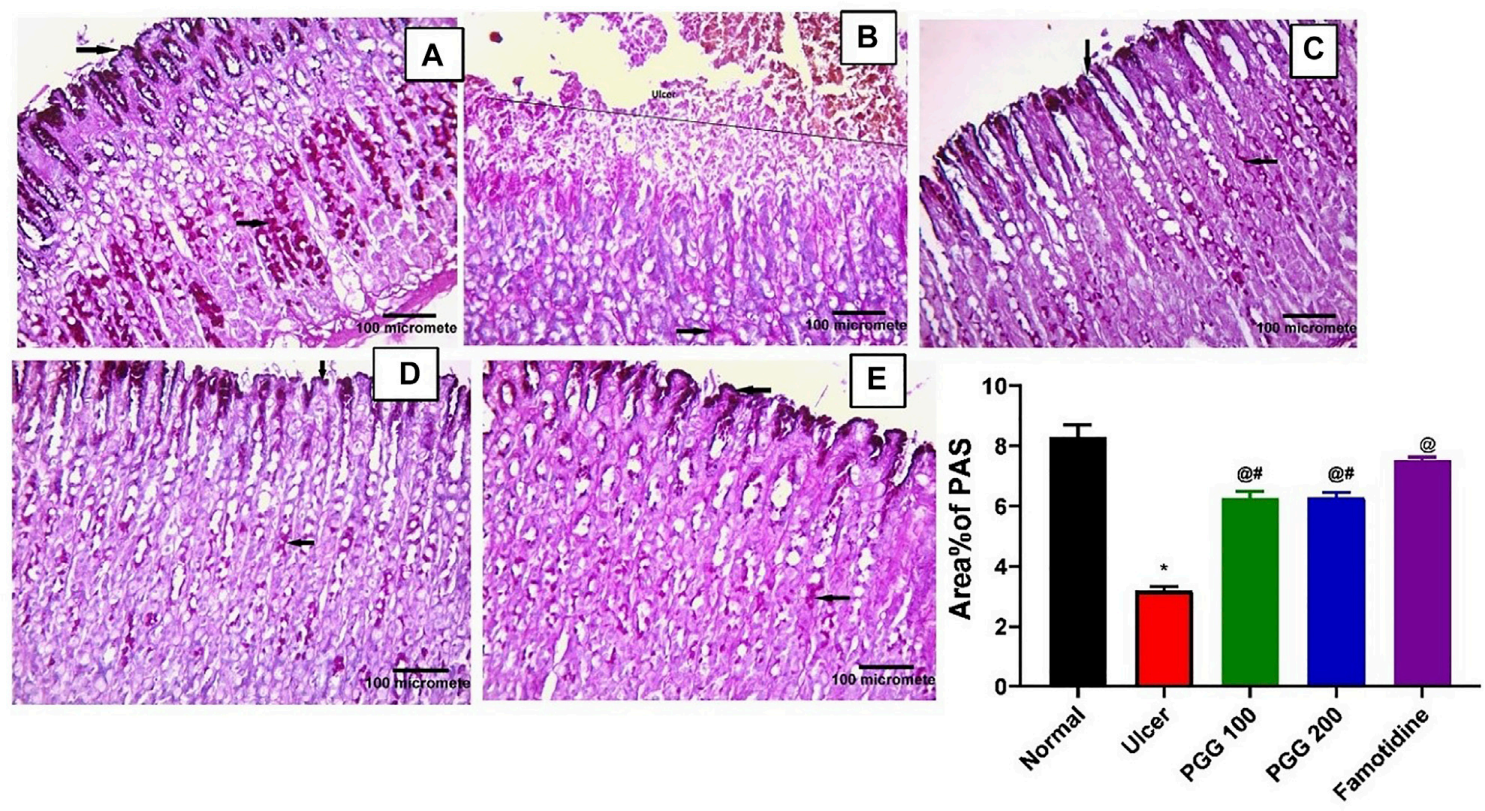
Pentagalloyl glucose (PGG) effects on the gastric glycoprotein content of the gastric mucosa of ulcerative rats. Photomicrographs of gastric sections stained by Periodic acid–Schiff stain (PAS) collected from **(A)** normal control group, **(B)** ulcerative animals (indomethacin group, 60 mg/kg), **(C,D)** indomethacin + PGG-treated groups (100 mg/kg, and 200 mg/kg, respectively), and **(E)** indomethacin + famotidine-treated group (10 mg/kg). [PAS × 200]. The bar graph showed quantitative analysis of area percentage of PAS staining. Results are expressed as mean ± SEM, *n* = 10. *^, @, #^significantly different compared to the control group and indomethacin and famotidine groups, respectively at *p* < 0.05 by one-way ANOVA followed by Tukey’s post hoc test.

Mucous secretion is thought to be an important protective mechanism for the stomach mucosa against lesions (Mahmoud et al., 2021). One of the most important mechanisms of local stomach mucosal protection is mucus formation. To investigate the mechanism of the anti-ulcer effect of PGG, gastric mucosal glycoproteins, an indicator of gastric mucous secretion, was detected using PAS staining. Both control or famotidine-treated groups showed strong PAS positive reaction on the mucosal surface down to the pits, isthmus, neck, and body regions of the gastric glands, indicating normal glycoprotein content of the gastric mucosa (Figures 2A,E). Indomethacin caused depletion of gastric mucosal glycoprotein content where complete loss of PAS reaction at the ulcerated region was observed, but some positive reaction in the remaining part of the gastric glands can be noticed (Figure 2B). PGG at both dose levels restored glycoprotein content of the gastric mucosa where both groups showed strong PAS positive reaction on the mucosal surface down to the pits, isthmus, neck, and body regions of the gastric glands (Figures 2C,D).

**FIGURE 3:**
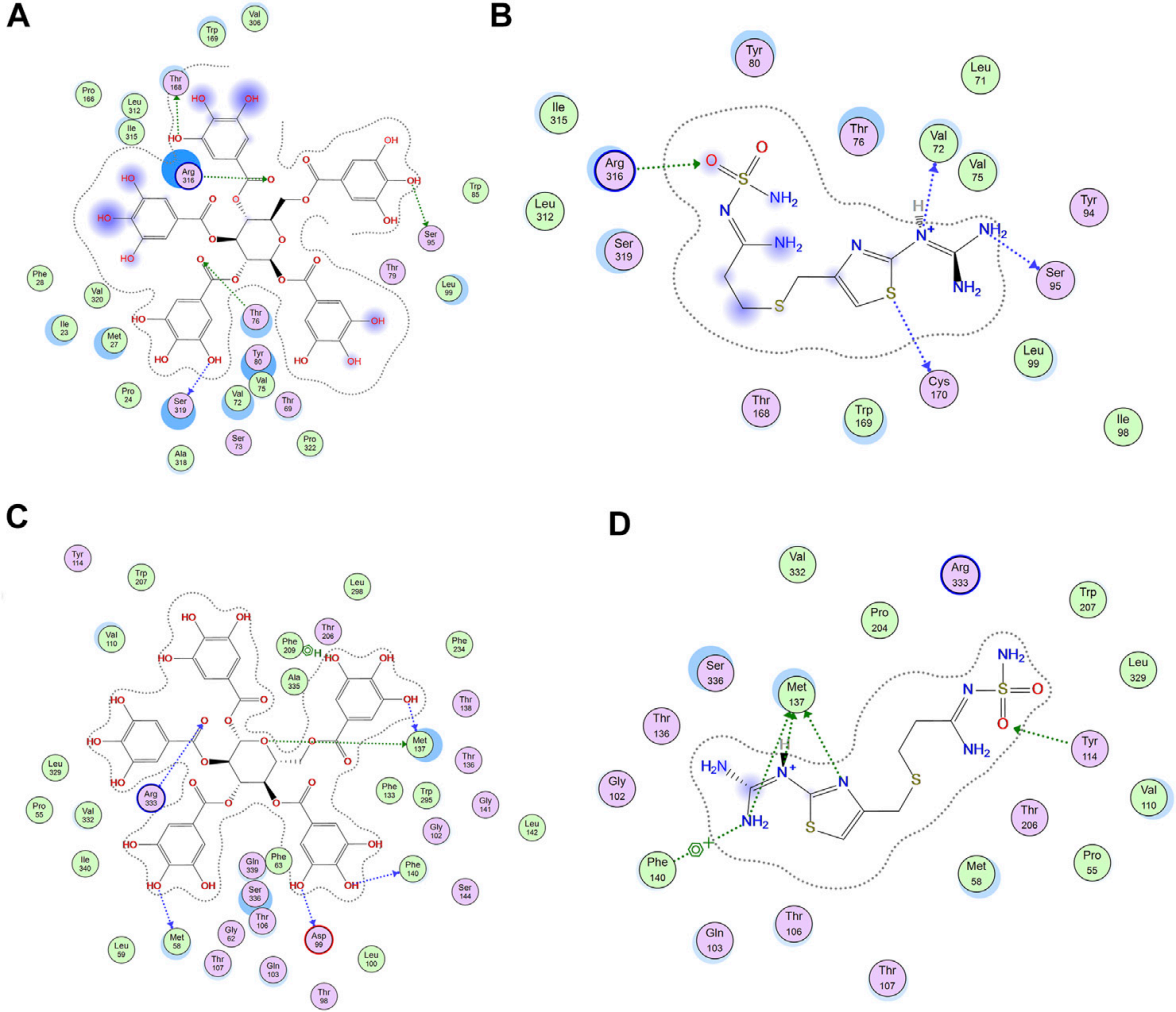
2D-interactions of **(A)** pentagalloyl glucose (PGG) and **(B)** famotidine with amino acid residues on EP4 and **(C)** PGG and **(D)** famotidine with amino acid residue EP3 receptor.

The increased mucin content by PGG is crucial for cytoprotective and ulcer healing effect of PGG. It was reported that indomethacin-induced delay in the healing of chronic gastric ulcers is reversed by 11-deoxy-PGE1 (EP3/EP4 agonist) (Hatazawa et al., 2007). Therefore, we virtually explored the interaction of PGG with two prostaglandin receptors, EP3 (PDB id: 6AK3) and EP4 (PDB id: 5YWY), following the procedure adopted for the molecular docking in our previous study (Mahmoud et al., 2021). PGG displayed low binding energy compared to the reference drug famotidine when docked into the active side of EP3 and EP4. It showed four crucial interactions with Met 137, Me58, Arg 333, and Phe 209 for EP3, while furnishing a special H-bonding interaction with Thr 168 in the case of EP4 along with four other interactions, Table 1 and Figure 3. Restoration of mucin content of gastric mucosa by PGG may be mediated by increasing PGE2 synthesis or activation of prostaglandin receptors, EP3 and EP4, as indicated by docking studies. Similar results were observed from other tannins, such as tellimagrandin II (score functions (kcal/mol) are -31.31 and -26.88 for EP3 and EP4, respectively) and casuarinin (score functions (kcal/mol) are −25.47 and −25.96 for EP3 and EP4, respectively) (Mahmoud et al., 2021).

**TABLE 1:** Scoring functions (kcal/mol) and amino acid interactions for PGG and famotidine docked to two receptors (EP4 and EP3).

Compound	EP4		EP3	
	Score	Interactions	Score	Interactions
PGG	−25.34	Thr 168 (H bonding)	−28.42	Met 137 (H bonding)
		Ser 319 (H bonding)		Asp 99 (H bonding)
		Ser 95 (H bonding)		Met 58 (H bonding)
		ARG 316 (H bonding)		Phe 140 (H bonding)
		Thr 76 (H bonding)		Met 137 (H bonding)
				Arg 333 (H bonding)
				Phe 209 (H-pi and H bonding)
Famotidine	−11.35	Cys 170 (H bonding)	−14.05	Met 137 (H bonding)
		Arg 316 (H bonding)		Tyr 114 (H bonding)
		Ser 95 (H bonding)		Phe 140 (H-pi)
		Val 72 (H bonding)		

### 3.3 Effect of PGG on Gastric Histological Changes

**FIGURE 4:**
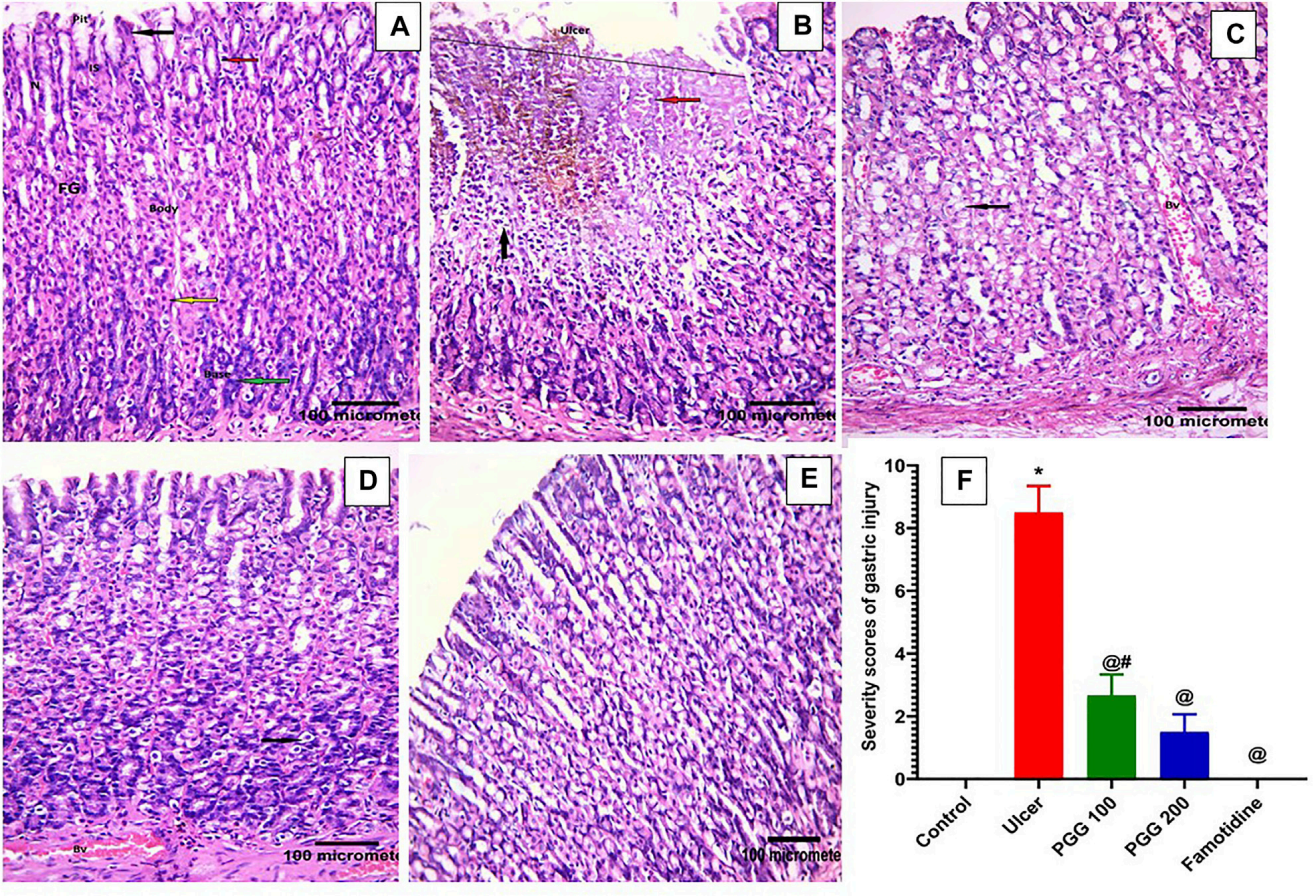
Histopathological effects of pentagalloyl glucose (PGG) effects on the stomach mucosal tissues of animals. Representative gastric sections from **(A)** normal control group, **(B)** ulcerative animals (indomethacin alone, 60 mg/kg), **(C)** indomethacin + PGG-treated groups (100 mg/kg), **(D)** indomethacin + PGG-treated groups (200 mg/kg), and **(E)** indomethacin + famotidine-treated group (10 mg/kg). **(F)** Column graph illustrates the severity scores of gastric injuries among groups, Data are presented as mean ± SEM, *^,@,#^ *p* < 0.05 vs. control, ulcer, and famotidine groups, respectively, by ANOVA followed by Dunnett’s test. Fundic glands (FGs), gastric pits (Pits), isthmus (IS), neck (N), columnar epithelial cells (black arrow), mucous neck cells (red arrow), pyramidal oxyntic cells (yellow arrow), peptic cells or vacuolation (green arrow), tissue erosion and degeneration (black arrow), exfoliated cells (red arrow), extravasated blood (orang arrow), cellular infiltration (circle), and congested blood vessels (BV), [H&E × 200].

To reveal whether PGG was able to reverse indomethacin-induced gastric structural alteration, a histopathological study was performed. This study showed that both normal control and indomethacin group pretreated with famotidine demonstrated normal histology of gastric tissue with gastric mucosa containing closely packed fundic glands that open in the luminal surface by gastric pits. These fundic glands occupy the whole thickness of the fundic mucosa extending from the gastric pits. Each gland is formed of the isthmus, neck, body, and base. The upper part of the fundic glands is lined with surface columnar epithelial cells with basal oval nuclei. Mucous neck cells have flat basal nuclei. Pyramidal oxyntic cells have central rounded nuclei and deep acidophilic cytoplasm. Peptic cells have basal oval nuclei and basophilic cytoplasm (Figures 4A,E). The indomethacin only–treated group showed a large mucosal ulcer affecting the upper half of the fundic glands. Tissue erosion and degeneration can be seen; the fundic glands lose their orientation and arrangement. Exfoliated cells appear in the lumen. Extravasated blood can be seen within the fundic glands. Some fundic gland cells appear. Inflammatory cellular infiltration was also observed (Total score: 8.50 ± 0.85) (Figure 4B). Indomethacin groups treated with 100 mg/kg or 200 mg/kg PGG showed dose-dependent, marked improvement of gastric mucosa with no ulcer. Some vacuolations and congested blood vessels may be seen (Total score: 2.67 ± 0.67 and 1.50 ± 0.56 respectively) (Figures 4C,D). These findings are in accordance with those of a previous study that showed that PGG at 100 and 200 mg mitigated aspirin-induced gastric histological changes in rats (Moharram et al., 2017).

### 3.4 Effect of PGG on Immunoexpression of Endothelial Nitric Oxide Synthase

**FIGURE 5:**
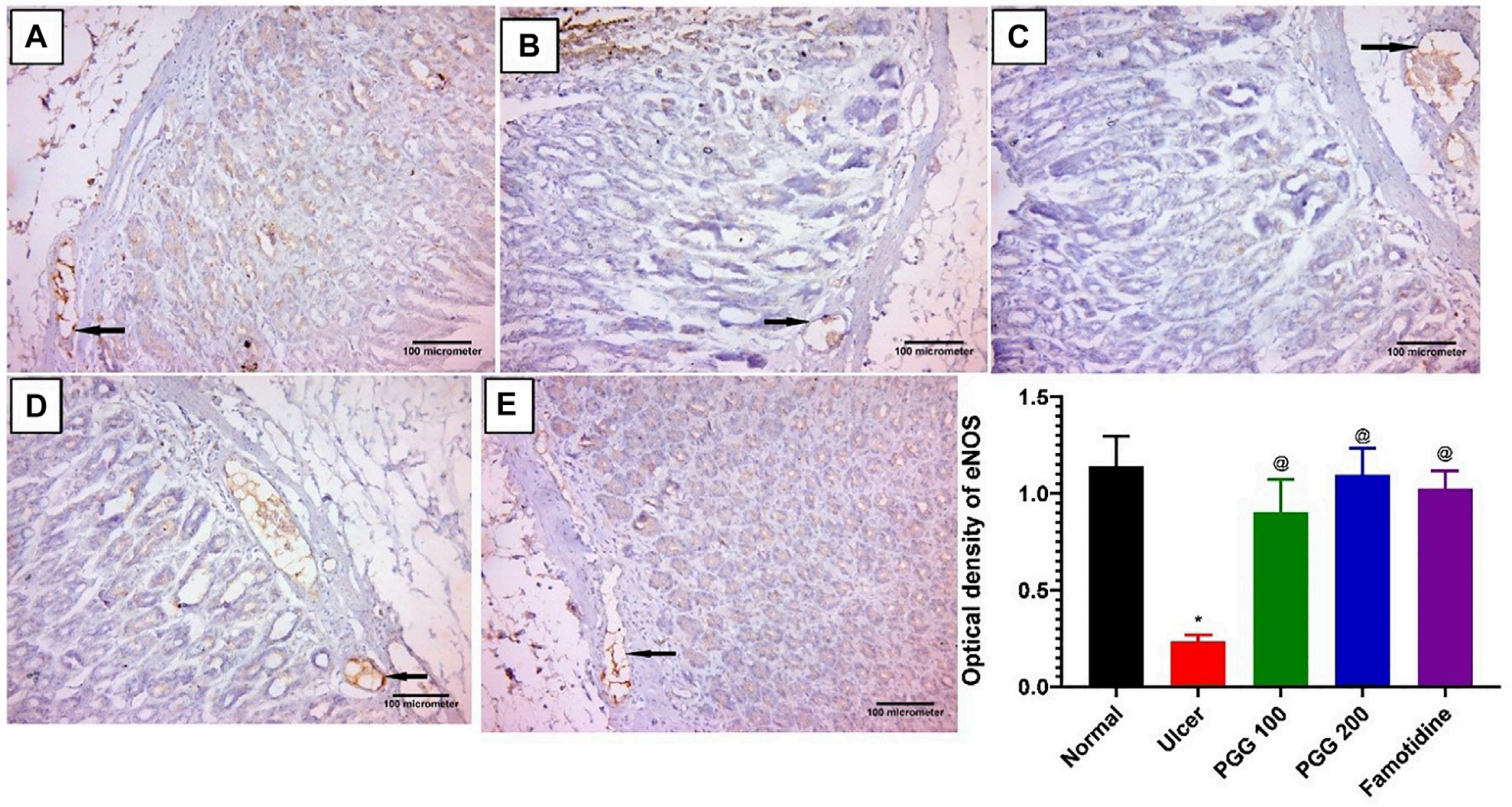
Immunohistochemical staining of endothelial nitric oxide synthase (eNOS) in gastric mucosal tissues. Representative gastric mucosal sections from **(A)** normal control animals, **(B)** ulcerative animals treated with indomethacin alone (60 mg/kg), **(C,D)** ulcerative rats treated with indomethacin + PGG at two doses: 100 and 200 mg/kg, respectively, and **(E)** ulcerative animals treated with indomethacin + famotidine-treated group (10 mg/kg). Brown and blue indicate positive and negative staining of eNOS, respectively. Avidine biotin peroxidase stained with Hx counter stain x200. Data are expressed as mean ± S.E, *n* = 10. *^,@^significantly different from corresponding control group of rats and indomethacin group, respectively, at *p* < 0.05 by one-way ANOVA was used for statistical analysis followed by Tukey’s post hoc test.

The action of nitric oxide in the stomach mucosa is paradoxical. Nitric oxide is made from l-arginine in a chemical reaction mediated by either cytotoxic-inducible nitric oxide synthase (iNOS) or cytoprotective constitutive eNOS (Abdel-Raheem, 2010). In this study, we investigated the effect of PGG on the nitric oxide signaling pathway and its contribution in the gastroprotective effect of PGG. As illustrated in Figure 5, indomethacin-treated rats exhibited a 6-fold dramatic decrease in eNOS level when related to the control group. Pretreatment with the reference drug famotidine or the tested compound PGG at doses (100 and 200 mg/kg) markedly increased eNOS levels by 4.4, 3.9, and 4.7 folds, respectively, when related to the ulcerative animals (indomethacin-treated group). The increased expression of eNOS by PGG may be responsible for the observed gastroprotective effects of PGG in the present study. NO produced by eNOS improves ulcer healing by increasing vasodilation, inducing angiogenesis, scavenging the toxic-free radicals, and reducing leukocyte infiltration, resulting in epithelial tissue integrity restoration and enhanced mucous secretion (Khattab et al., 2001; Abd El-Rady et al., 2021), as observed in our study.

### 3.5 Effect of PGG on Immunoexpression of Inducible Nitric Oxide Synthase

**FIGURE 6:**
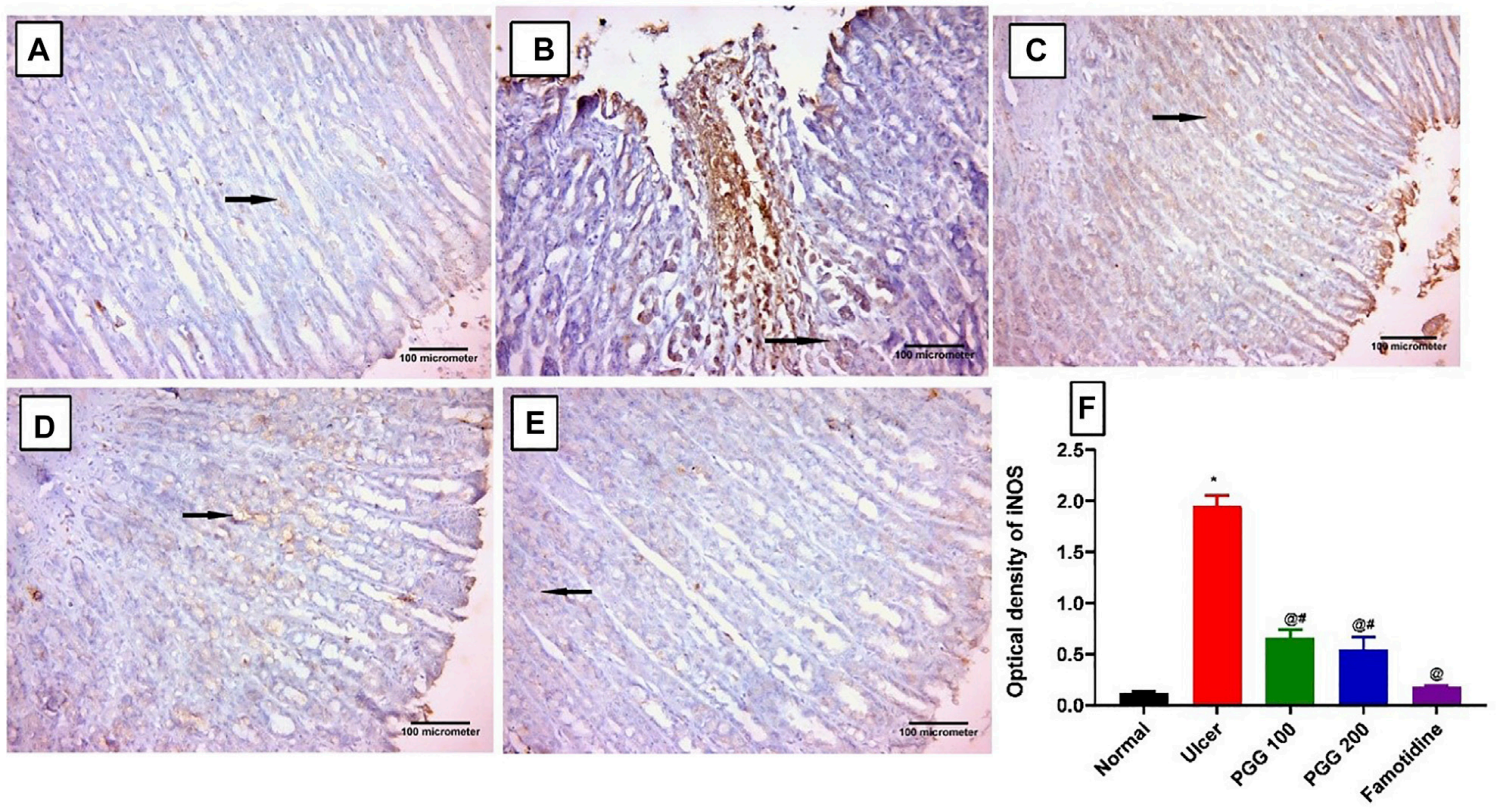
Immunohistochemical staining of inducible nitric oxide synthase (iNOS) in gastric mucosal tissues. **(A)** Control and **(E)** famotidine-pretreated ulcer groups furnished a minimal positive cytoplasmic immunoreactivity to iNOS in the gastric mucosal cells (arrows). **(B)** Ulcerative animals (indomethacin group) displayed an intense positive immune reaction to iNOS in the gastric mucosal cells (arrows). **(C,D)** PGG -pretreated ulcer groups (100 or 200 mg/kg) showed a reduction in iNOS immunoreactivity (arrows). iNOS immunostaining (avidine biotin peroxidase stained with Hx counter stain X 200, scale bar = 100 µm). **(F)** Quantitative analysis of immunoreactivity intensity of iNOS. Results are shown as mean ± SEM, *n* = 10. * ^@, #^significantly different compared the control group, indomethacin, and famotidine groups, respectively, at *p* < 0.05 by one-way ANOVA followed by Tukey’s post hoc test.

Nitric oxide produced by iNOS plays a role in ulcer formation through the production of cytotoxic peroxide–free radicals (Pan et al., 2005). We showed previously that indomethacin administration is associated with increased expression of gastric iNOS, and its inhibition promotes ulcer healing effects (Nabil et al., 2021). In this study, control and famotidine-pretreated ulcer group sections displayed a lower positive cytoplasmic immunoreactivity to iNOS in the gastric mucosal cells (Figures 6A,E). On the other hand, indomethacin-induced ulcer group sections demonstrated an intense and significant positive immune reaction to iNOS in the gastric mucosal cells when related to the control group (*p* < 0.05) (Figure 6B). The PGG-treated groups at both dose levels revealed a significant reduction in iNOS immunoreactivity when compared to the ulcerative animals, indomethacin group (*p* < 0.05) (Figures 6C,D, respectively). Moreover, famotidine exerted more potent effects than PGG in decreasing iNOS expression, *p* < 0.05, (Figure 6F). It was observed that PGG lowered the expression levels of iNOS in LPS-stimulated macrophages (Zhang et al., 2021); this confirms its anti-inflammatory and cytoprotective effects observed in the current study.

### 3.6 Effect of PGG on Oxidative Stress

**FIGURE 7:**
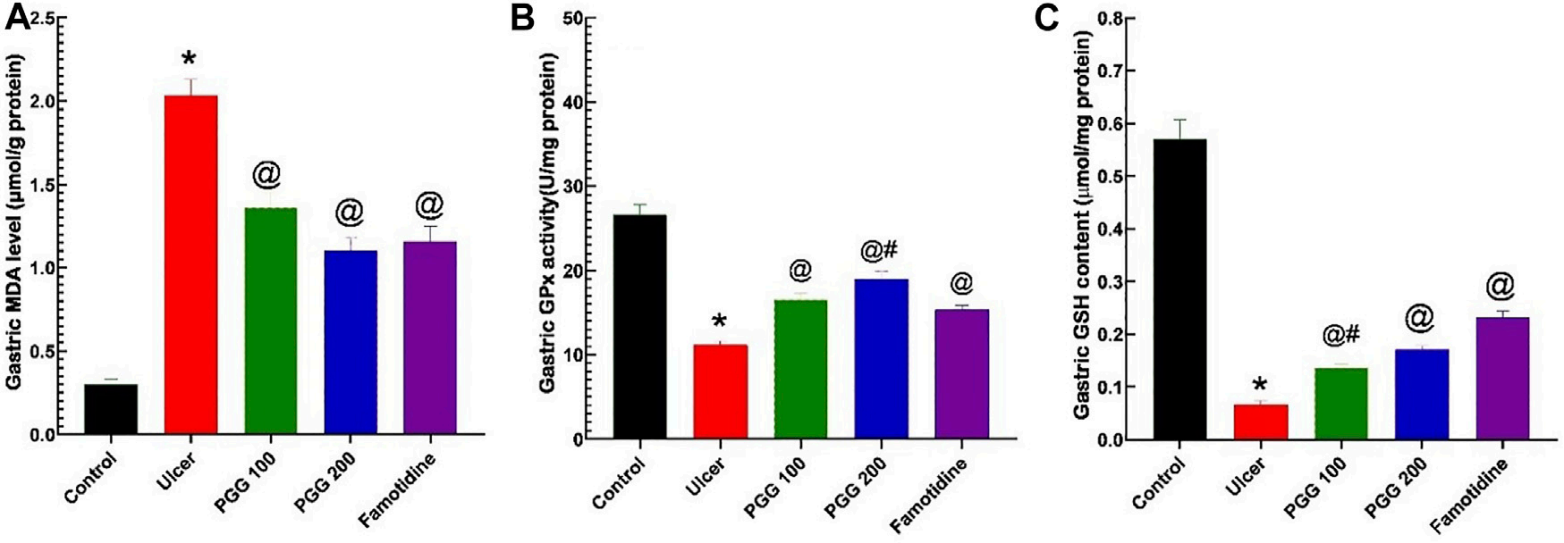
Results from the malondialdehyde (MDA) level **(A)**, glutathione peroxidase activity (GPx), **(B)** and reduced glutathione content (GSH) **(C)** in gastric tissues. The data are shown as the mean ± SEM (*n* = 6). *, ^@^, ^#^Mean values with different symbols are significantly different (*p* < 0.05) according to one-way ANOVA followed by Tukey’s post hoc test vs. control, indomethacin ulcer, or famotidine groups, respectively.

In animal studies, reactive oxygen species, ROS, produced by activated neutrophils in gastric tissues are linked to the etiology and delayed stomach ulcer repair caused by indomethacin (Wang et al., 2014). Intracellular antioxidant systems, such as GPx, GSH, SOD, and catalase, can neutralize the produced ROS (Antonisamy et al., 2016). To further investigate the mechanism of action of PGG to prevent indomethacin-induced gastric injury, the endogenous antioxidants, such as GPx activity and GSH content, were examined in gastric tissues. Furthermore, malondialdehyde (MDA), a lipid peroxidation product resulted from increased cellular oxidative stress, was also measured. Figure 7 shows that MDA content was elevated in indomethacin group administration (*p* < 0.05, Figure 7A), while GPX activity and GSH content were decreased (*p* < 0.05, Figures 7B,C, respectively) compared with the control group. PGG at both doses or famotidine pretreatment exerted protective effects, in which MDA content was decreased and GPX activity and GSH content were elevated in gastric tissues. Both PGG at the two dose levels and famotidine exerted similar protective effects on MDA, while the effect of PGG at 200-mg/kg level was stronger than that of famotidine on gastric GPX activity. Both 200 mg/kg PGG and famotidine had more potent effect on gastric GSH than PGG 100 mg/kg. These data indicated that PGG improved antioxidant capacity in the gastric injury model.

### 3.7 Effect of PGG on Gastric Inflammation

**FIGURE 8:**
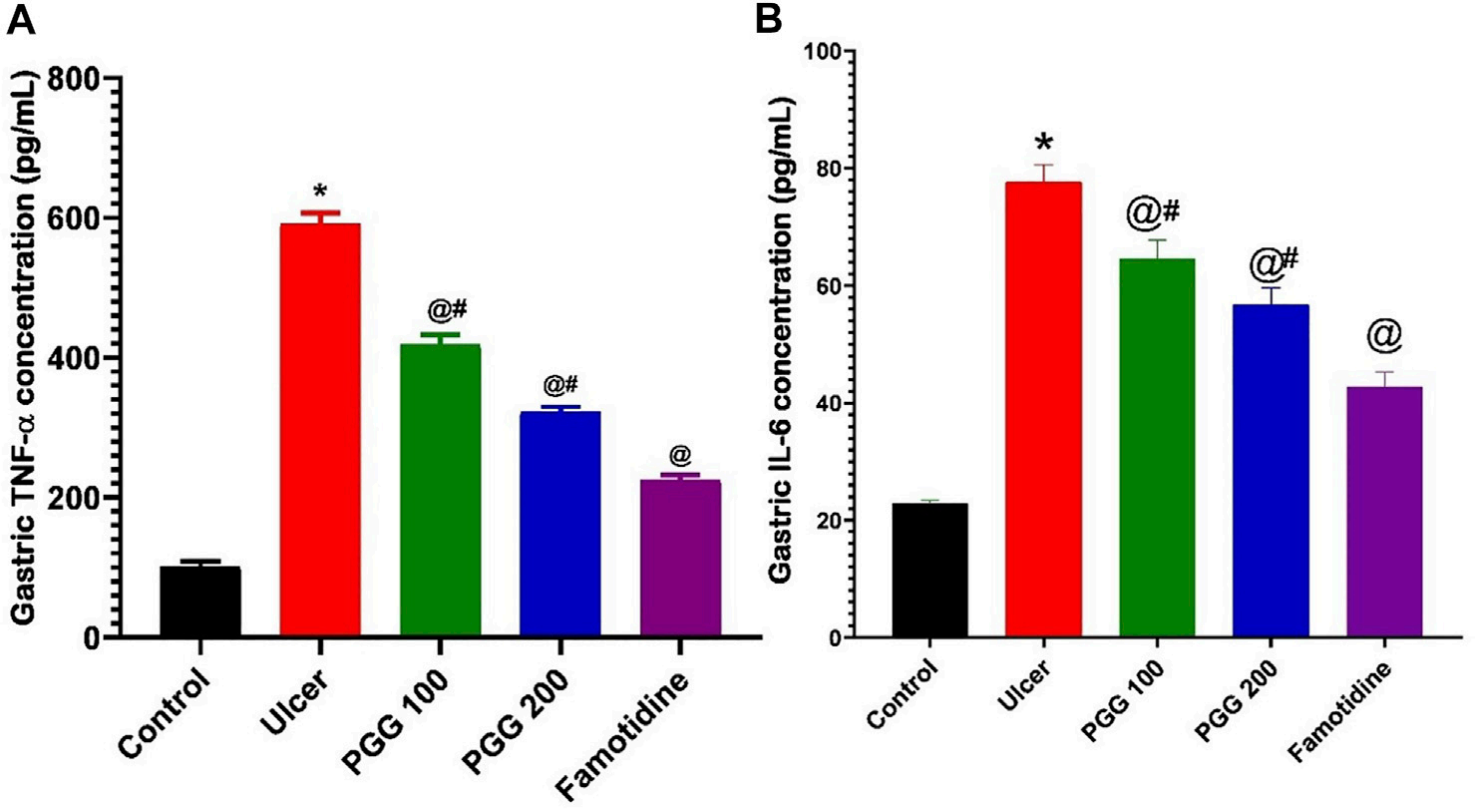
Effect of pretreatment with either pentagalloyl glucose (PGG) (100 or 200 mg/kg) or famotidine on gastric TNF-α levels **(A)** and IL-6 **(B)** of indomethacin-induced gastric injury. Data are expressed as mean ± SEM, *n* = 4. *, ^@^ and ^#^statistically significant from control, indomethacin group, or famotidine groups of rats, respectively, at *p* < 0.05 by one-way ANOVA was used for statistical analysis followed by Tukey’s post hoc test.

The induction and healing of gastric ulcers are influenced by a variety of inflammatory variables (Miyata et al., 1997; Mahmoud et al., 2021). As shown in the current study, indomethacin caused infiltration of inflammatory cells to the gastric tissues with subsequent release of ROS and inflammatory mediators. We previously reported that both TNF-α and IL-6 were elevated in gastric tissues following indomethacin administration (Mahmoud et al., 2021; Nabil et al., 2021). TNF-α and IL-6 are proinflammatory cytokines that play a role in the development of stomach ulcers and inflammation (Aziz et al., 2019). TNF-α induces the subsequent production of additional inflammatory factors, such as IL-2 and IL-6, that could activate neutrophils and release oxygen-free radicals, generate acute phase proteins, obstruct gastric mucosal blood microcirculation, and increase gastric mucosal injury (Su et al., 2017; Lian et al., 2020). In the current study, the levels of inflammatory factors TNF-α and IL-6 were also significantly elevated in gastric injury rats induced by indomethacin (*p* < 0.05; Figure 8). In rats pretreated with either PGG (100, 200 mg/kg) or famotidine, a decrease in inflammatory factors was observed, indicating that PGG efficiently relieved inflammation. The decrease was clearer in the famotidine group. In this context, PGG was able to suppress the expression of proinflammatory cytokines, including IL-1β and TNF-ɑ, and inflammatory enzyme, COX-2, in advanced glycation end product (AGE)–treated renal mesangial cells (Tong et al., 2021). Moreover, in LPS-stimulated RAW 264.7 cells, PGG dramatically reduced secretions of TNF-α, IL-1, IL-6, and NO (Zhang et al., 2021). Furthermore, in accordance with the present study, *in vivo* studies on the animal model of aortic aneurysm showed that PGG nanoparticles suppressed inflammatory cell infiltration and systemic inflammation (Wang et al., 2021).

### 3.8 Effect of PGG on Platelet Endothelial Cell Adhesion Molecule-1

**FIGURE 9:**
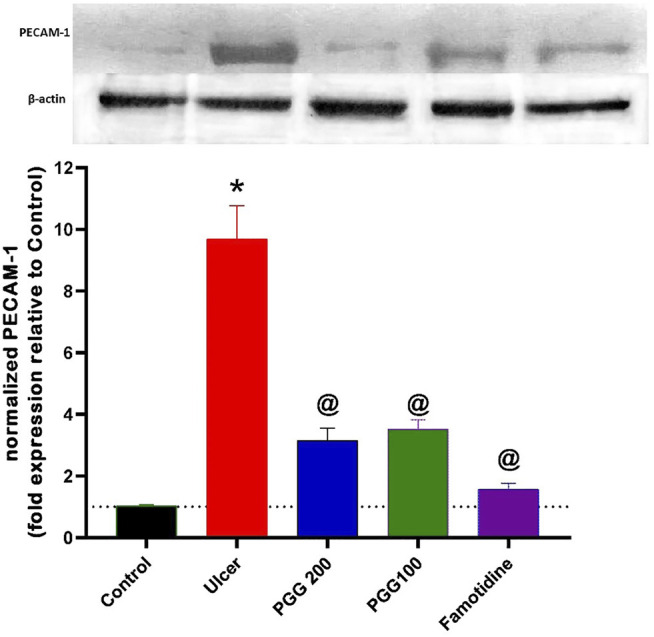
Upper panel represents the effect of pretreatment with either pentagalloyl glucose (PGG) (100 or 200 mg/kg) or famotidine on gastric PECAM-1 level of indomethacin-induced gastric injury. The lower panel represents densitometry analysis of the ratio of PECAM-1 protein over β-actin protein. Data are expressed as mean ± SEM, *n* = 3. *, ^@^ Statistically significant from control and indomethacin groups of rats, respectively, at *p* < 0.05 by one-way ANOVA was used for statistical analysis followed by Tukey’s post hoc test.

PECAM-1 (platelet endothelial cell adhesion molecule-1 or cluster of differentiation 31, CD31) is a 130-kDa Ig superfamily member that is expressed on platelets and leukocytes and is particularly concentrated at endothelial cell–cell junctions (Newman, 1999). It was reported that increased expression of PECAM-1 is associated with increased leukocyte trans-endothelial migration and subsequent inflammation (Schenkel et al., 2004). PECAM-1 is engaged in the trans-endothelial migratory process in both leukocytes and endothelial cells (Muller et al., 1993). It was reported that PECAM-1 antibody administration attenuated lung inflammation and minimized tissue necrosis and leukocyte infiltration in a model of myocardial infarction (Vaporciyan et al., 1993; Gumina et al., 1996). PECAM-1’s role, however, has not been explored in any experimental model of indomethacin-induced gastric ulcer. As shown in Figure 9, indomethacin administration was associated with increased expression of PECAM-1 by 8.4 fold, which explains the increased leukocyte infiltration observed in the histopathological study and the increased levels of proinflammatory cytokines, TNF- and IL-6, in the gastric tissues of the ulcer group. This finding is in accordance with that of a previous study by Konturek et al. (2004), who showed that PECAM-1 is overexpressed in vehicle-treated gastric mucosa at the ulcer margin, and capsaicin attenuated this effect in acetic acid–induced gastric ulcer. However, a previous study of Alese et al. (2017) showed that PECAM-1 is decreased in aspirin-induced gastric ulcer, and flavonoid fraction of *Musa paradisiaca* increased its level. The difference in results may be attributed to the difference in the experimental design. As shown in Figure 9, the present study revealed that both PGG (100 & 200 mg/kg) and famotidine administration attenuated increased PECAM expression (6.4, 6.7, and 8.3 folds, respectively) compared to the ulcer group. The present study confirmed that PECAM-1 plays an important role in the ulcerogenic effect of indomethacin, and inhibition of its expression by PGG and famotidine may mediate their antiulcerogenic effects. Further studies using antibodies against PECAM-1 are required to confirm its role in indomethacin-induced ulcers.

### 3.9 Effect of PGG on Vascular Endothelial Growth Factor

**FIGURE 10:**
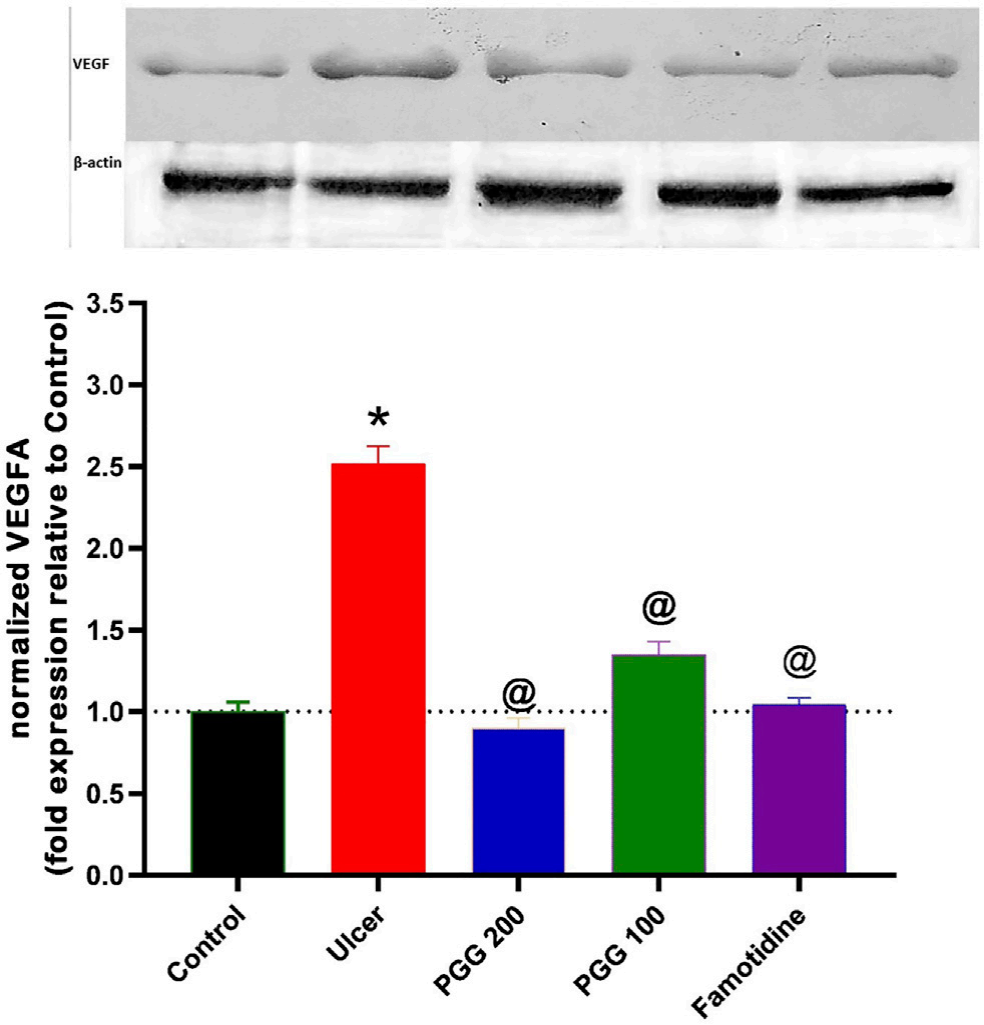
Upper panel represents the effect of pretreatment with either pentagalloyl glucose (PGG) (100 or 200 mg/kg) or famotidine on gastric VEGF level of indomethacin-induced gastric injury. The lower panel represents densitometry analysis of the ratio of VEGF protein over β-actin protein. Data are expressed as mean ± SEM, *n* = 3. * and @ Statistically significant from control or indomethacin groups of rats, respectively, at *p* < 0.05 by one-way ANOVA was used for statistical analysis followed by Tukey’s post hoc test.

As shown in Figure 10, indomethacin-induced ulcer was associated with increased expression of VEGF by 150% when compared to the control group. Administration of either PGG (100 and 200 mg/kg) or famotidine decreased VEGF expression by 46, 64, and 58.4% when compared to the ulcer group. Vascular endothelial factor is an important growth factor that is involved in angiogenesis and ulcer healing processes. Our results indicate that indomethacin increased VEGF in gastric tissues in an attempt to start ulcer healing following gastric mucosal damage. As a result of PGG and famotidine, there may be less injury to the gastric mucosa, resulting in VEGF synthesis not being stimulated. These findings are confirmed by the previous study in which 50% ethanol administration caused deep necrosis in the gastric mucosa and increased VEGF expression after 3 h of ethanol administration, and *Punica granatum* peel extract administration attenuated this increase (Jones et al., 1999). On the contrary, other studies showed that indomethacin administration was associated with decreased expression of VEGF, and *Chasmanthera dependens* extract or cimetidine increased it (Tijani et al., 2021). However, the experimental design is different where drugs are used for 14 days following ulcer induction. In our study, drugs were used 8 days before indomethacin administration, and VEGF expression was measured 6 h after indomethacin administration, not after 14 days as in the previous study.

## 4 Conclusion

This study is the first to show that pentagalloyl glucose (PGG) has a protective effect in a rat model of indomethacin-induced gastric ulcer. PGG’s cytoprotective effect could be linked to an increase of gastric mucosal mucopolysaccharides, reduction of oxidative stress, triggered inflammatory response, and modulating NO/eNOS/iNOS signaling. The proven therapeutic benefits of PGG in the present study suggest that it could be effective in the treatment or prevention of gastric ulcers. However, further experiments are recommended to confirm the obtained results in other ulcer models and to evaluate the safety of PGG and determine the therapeutic doses and time.

## Data Availability

The original contributions presented in the study are included in the article, further inquiries can be directed to the corresponding author.

## Abbreviations

eNOS: endothelial nitric oxide synthase; GPx: glutathione peroxidase; GRO: growth-regulated oncogene; GSH: reduced glutathione; IL-6: interleukin 6; iNOS: inducible nitric oxide synthase; MAPK1: mitogen-activated protein kinase 1; MDA: malondialdehyde; NMR: nuclear magnetic resonance; NSAIDs: non-steroidal anti-inflammatory drugs; PECAM-1: platelet endothelial cell adhesion molecule-1; PGG: pentagalloyl glucose; SDS-PAGE: sodium dodecyl sulfate-polyacrylamide gel electrophoresis; SOD: superoxide dismutase; TNF-α: tumor necrosis factor; VEGF: vascular endothelial growth factor.
